# Plasma metabolomics of the time resolved response to *Opisthorchis felineus* infection in an animal model (golden hamster, *Mesocricetus auratus*)

**DOI:** 10.1371/journal.pntd.0008015

**Published:** 2020-01-24

**Authors:** Daria Kokova, Aswin Verhoeven, Ekaterina A. Perina, Vladimir V. Ivanov, Elena M. Knyazeva, Irina V. Saltykova, Oleg A. Mayboroda

**Affiliations:** 1 Department of Parasitology, Leiden University Medical Center, Leiden, The Netherlands; 2 Laboratory of clinical metabolomics, Tomsk State University, Tomsk, Russia; 3 Center for Proteomics and Metabolomics, Leiden University Medical Centre, Leiden, The Netherlands; 4 Central Research Laboratory Siberian State Medical University, Tomsk, Russian Federation; 5 School of Core Engineering Education, National Research Tomsk Polytechnic University, Tomsk, Russian Federation; Queen's University Belfast, UNITED KINGDOM

## Abstract

**Background:**

Opisthorchiasis is a hepatobiliary disease caused by flukes of the trematode family *Opisthorchiidae*. Opisthorchiasis can lead to severe hepatobiliary morbidity and is classified as a carcinogenic agent. Here we investigate the time-resolved metabolic response to *Opisthorchis felineus* infection in an animal model.

**Methodology:**

Thirty golden hamsters were divided in three groups: severe infection (50 metacercariae/hamster), mild infection (15 metacercariae/hamster) and uninfected (vehicle-PBS) groups. Each group consisted of equal number of male and female animals. Plasma samples were collected one day before the infection and then every two weeks up to week 22 after infection. The samples were subjected to ^1^H Nuclear Magnetic Resonance (NMR) spectroscopy and multivariate statistical modelling.

**Principal findings:**

The time-resolved study of the metabolic response to *Opisthorchis* infection in plasma in the main lines agrees with our previous report on urine data. The response reaches its peak around the 4th week of infection and stabilizes after the 10th week. Yet, unlike the urinary data there is no strong effect of the gender in the data and the intensity of infection is presented in the first two principal components of the PCA model. The main trends of the metabolic response to the infection in blood plasma are the transient depletion of essential amino acids and an increase in lipoprotein and cholesterol concentrations.

**Conclusions:**

The time resolved metabolic signature of *Opisthorchis* infection in the hamster’s plasma shows a coherent shift in amino acids and lipid metabolism. Our work provides insight into the metabolic basis of the host response on the helminth infection.

## Author summary

Opisthorchiasis is a parasitic infection caused by liver flukes of the *Opisthorchiidae* family. The liver fluke infection triggers development of hepatobiliary pathologies such as chronic forms of cholecystitis, cholangitis, pancreatitis, and cholelithiasis and increases the risk of intrahepatic cholangiocarcinoma. This manuscript is the second part of our outgoing project dedicated to a comprehensive description of the metabolic response to opisthorchiasis (more specifically *Opisthorchis felineus*) in an animal model. We show that the metabolic response in blood plasma is unfolding according to the same scenario as in urine, reaching its peak at the 4th week and stabilizing after the 10th week post-infection. Yet, unlike the response described in urine, the observed metabolic response in plasma is less gender specific. Moreover, the biochemical basis of the detected response in blood plasma is restricted to the remodeling of the lipid metabolism and the transient depletion of essential amino acids. Together with our first manuscript this report forms the first systematic description of the metabolic response on opisthorchiasis in an animal model using two easily accessible biofluids. Thus, this contribution provides novel results and fills an information gap still existing in the analytically driven characterization of the “Siberian liver fluke”, *Opisthorchis felineus*.

## Introduction

Opisthorchiasis is a hepatobiliary disease caused by flukes of the trematode family *Opisthorchiidae*: *Opisthorchis felineus* (*O*. *felineus*), *Opisthorchis viverrini* (*O*. *viverrini*) and *Clonorchis sinensis* (*C*. *sinensis*). Together they affect more than 45 million people and 600–750 million people are at risk in the endemic regions of Eurasia [1]. The clinical manifestations of acute opisthorchiasis are non-specific and its chronic stages are often asymptomatic [2, 3]. Yet, not only does the chronic opisthorchiasis leads to severe hepatobiliary morbidities [2, 4], but also *O*. *viverrini* and *C*. *sinensis* were classified as carcinogenic agents by the International Agency for Research on Cancer (IARC) [5]. Consequently, the application of modern analytical technologies to the study of the parasite and its interaction with the host are becoming an essential element in the search for the mechanisms of the hepatobiliary morbidity.

Over the last three decades each of four key levels of biological regulations, namely genome, transcriptome, proteome and metabolome were strengthened by the corresponding “omics” disciplines. To date, a draft of the genome of the *O*. *viverrini* has been published [6, 7], the transcriptome data have been available for some time [8] and despite a rather critical assessment of the proteome data [9] there are 160 tegumental and 43 excreted proteins described [10]. The data on metabolomics of the *Opisthorchiidae* and host responses to the infection are still very limited. For an overview of the current status, advantages and limitations of metabolomics in helminthology we refer to our recent critical review of the topic [11]. Here we only would like to mention two factors that contribute to a slower growth of available helminth-related metabolomics data: the chemical diversity of the metabolites leading to an increased complexity of the analytical workflows and the difficulties with a straight-forward mechanistic interpretation of the data (especially data on body fluids). Yet, as a post-genomic discipline, metabolomics bids a strong alternative to conventional biochemical experimentation by combining advanced analytical techniques capable of simultaneously detecting multiple compounds with multivariate modeling and/or machine learning algorithms. This way the physiological status of an organism can be represented as a combination of metabolites concentrations/abundances in biofluids.

The present study is the second part of our work on the metabolomics of the host response to the *O*. *felineus* infection. The “Siberian liver fluke”, *O*. *felineus* [12] forms a health risk for a population that is similar in size to the population in the endemic region of *O*. *viverrini* [13]. It is also known for its cancerogenic properties but remains far less studied. Of the four levels of biological regulation (genome, transcriptome, proteome, and metabolome) only a single report on the transcriptome of the different life stages of the parasite has been published so far [14]. To fill the existing data gap, we concentrate on a comprehensive description of the time-resolved response of the host on the *O*. *felineus* infection using metabolomics. The first part was dedicated to the metabolic changes detected in the urine samples [15], the current work covers the infection-dependent changes in the blood plasma samples. The study was designed as a longitudinal study with two degrees of the infection intensity. Both sets of samples (urine and plasma) were collected from the same animal models.

## Methods

### Ethics statement

All procedures with animals were carried out according to the recommendations of the national guidelines for animal caring: 12.08.1977 N 755 “On measures to further improve the organizational forms of work using experimental animals” and approved by the Siberian State Medical University (license number 3296 issued on 29.04.2013).

### Experimental opisthorchiasis model

The study design was described in detail the previously published urinary NMR (Nuclear Magnetic Resonance) based metabolomics study of experimental opisthorchiasis in the golden hamster (*Mesocricetus auratus*). Briefly, hamsters were divided in 3 groups: severe infection (50 metacercariae/hamster), mild infection (15 metacercariae/hamster) and uninfected (vehicle-PBS) groups. Each group consisted of 10 animals–five males and five females. The hamsters were approximately five weeks old at the time of infection.

The blood samples were collected from anesthetized hamsters one day before infection and then every two weeks, specifically at the weeks 2, 4, 6, 8, 10, 12, 14, 16, 18, 20 and 22 after infection according to the previously described protocol [16]. In brief, at each time point the hamsters were lightly restrained by gentle handling, the gingival vein was pierced with a 26-gauge needle, and blood was collected into test tubes pre-coated with sodium heparin. The hamsters were observed after each blood collection. The total volume of collected blood was selected to represent the maximal blood volume that can be withdrawn in accordance with ethical recommendations [17].

After collection into individually labelled Eppendorf tubes, all samples were transferred to a freezer (-80°C) for long term storage. The worm burden and egg output were described in details in the first part of the exploratory metabolomics of the experimental opisthorchiasis in a golden hamster [15].

### Eggs and worms counts

The method of calculation of the number of eggs per gram of feces and the estimation of the worm burden were described in the urinary NMR-based metabolomics study of experimental opisthorchiasis in the golden hamster (*Mesocricetus auratus*) [15].

### Blood plasma sample preparation and NMR data acquisition

All chemicals used for the buffer solution were purchased from Sigma-Aldrich except for the ^2^H_2_O which was purchased from Cortecnet and the 3-(trimethylsilyl)propionic-2,2,3,3-d^4^acid sodium salt (TSP) which was obtained from Cambridge Isotope Laboratories Inc. 96-well plates and NMR tubes were purchased from Bruker Biospin Ltd. (Germany).

The 33 μl of each plasma sample were mixed with 77 μl milli-Q water, then 110 μl buffer solution in H_2_O/D_2_O (80/20) with a pH of 7.4 containing 6.15 mM NaN_3_ and 4.64 mM TSP were added using a Gilson 215 liquid handler in combination with a Bruker SampleTrack system. Finally, 190 μl of each plasma-buffer mixture were transferred to 3 mm SampleJet NMR tubes and placed in refrigerated racks (6°C) in a Bruker SampleJet system until the NMR measurement.

NMR data were recorded using a Bruker 14.1T AVANCE II spectrometer (^1^H Larmor frequency 600 MHz), equipped with a triple resonance inverse cryoprobe with Z-gradient system and automatic tuning and matching. All NMR experiments were performed as described elsewhere [18] with some minor modifications: for the 1D nuclear Overhauser enhancement Spectroscopy (NOESY) and 1D Carr–Purcell–Meiboom–Gill pulse sequence (CPMG) experiments were collected with 32 scans; J-resolved spectra (JRES) were recorded with a total 2 scans for each increment in the indirect dimension. In addition, to achieve more information about the lipoprotein profile, a 1D diffusion-edited pulse sequence with diffusion time of 120 ms was performed [19]. After applying 4 dummy scans, a total of 98,304 data points covering a spectral width of 18,029 Hz and an exponential window function was applied with a line broadening factor of 1.0 Hz before Fourier transformation were accumulated using 16 scans for each sample.

### Spectral data processing and data analysis

Pre-processing of NMR data was performed in the KIMBLE workflow [20]. All ^1^H 1D NMR spectra were baseline-corrected using a polynomial fit of degree 5.

For exploratory analysis, the 1D NOESY spectra were binned from 0.5 to 9.5 ppm using adaptive intelligent binning [21]. Initial bin width was set to 0.02 ppm and final variable bins sizes were calculated based on the peaks edges in the spectra by using a lowest standard deviation criterion. The spectral region that is dominated by the residual water signal (4.35–5.0 ppm) was excluded from the data. The final data consisted of 469 bins of variable size × 343 observations (samples), which were normalized by PQN normalization to correct for dilution differences from sample to sample [22].

Principal component analysis (PCA) was performed on the univariate scaled NOESY binned dataset in the R statistical environment (http://www.r-project.org/, R versions 3.4.4), “Rcpm”, “pcaMethods” packages were used. The visualizations were made using “ggplot2”, “cowplot”, “caret” and “gridExtra” packages.

For supervised statistical modelling ANOVA-simultaneous component analysis (ASCA) was chosen. This method is based on the assumption that every variable is a function of all factors included in a study and uses analysis of variance for the analysis of the individual experiment factors and their interactions [23]. ASCA is specifically suited for time-resolved multigroup, multisubject and multivariate data. However, it is very sensitive to unbalanced design. 4.7% of all samples were lost during NMR acquisition because the spectra did not satisfy the quality inspection. Unfortunately, this resulted in unbalanced data. In order to apply ASCA to the data, imputation was performed. The nonparametric missing value imputation was performed using the Random Forest approach in the “missForest” package in the R environment [24].

ASCA was performed on the centered imputed dataset of NOESY experiment in Matlab R2016a (The MathWorks) [23]. The model quality was estimated by running a permutation test (number of permutations = 1000).

For selected small metabolites a quantification approach was applied which combines the superior signal-to-noise ratio of the CPMG pulse sequence with the superior resolution of the 2D JRES experiment [18, 20].

The multivariate analysis of variance was performed in R statistical environment (http://www.r-project.org/, R versions 3.4.4) using standard commands.

Diffusion-edited spectra were used for definition of the lipoprotein profile of plasma samples. For this aim, selected regions of the spectra were integrated after local baseline correction.

### Identification of the metabolites

Identification of metabolites was performed by exhausting search of the total 1D and 2D JRES data using the proprietary Bbiorefcode (Bruker Biospin Ltd.) database. Lipoproteins were identified using information performed R. Mallol at el. [25].

## Results

### The exploratory analysis of plasma metabolic profiles

**Fig 1:**
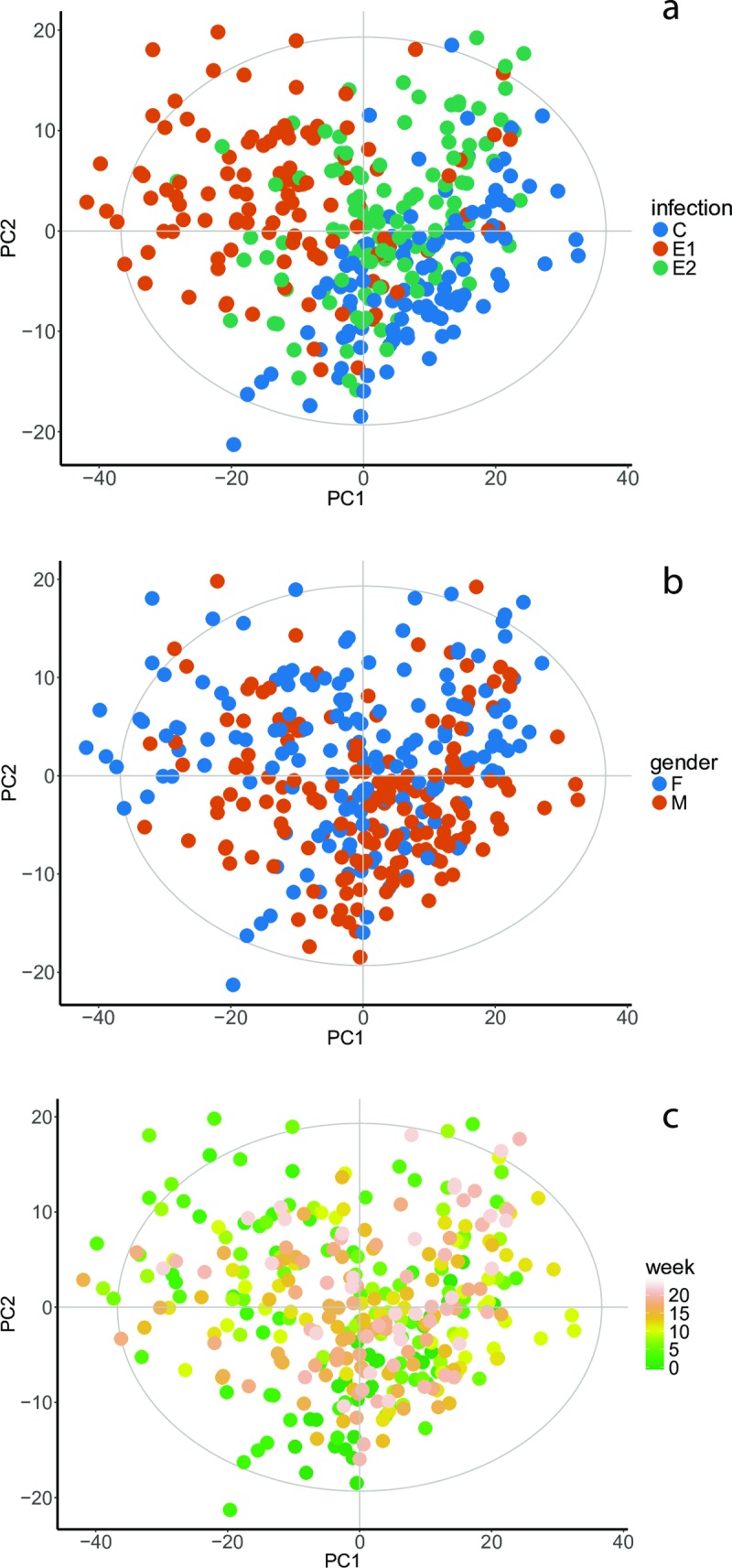
The scores plots of the PCA built on the entire dataset. Every block of the figure represents the same model colored according to the different experimental factors: infection status (**a**): C–control group, E1 –infected 50 metacercaria per animal, E2 –infected 15 metacercaria per animal; a gender (**b**): F–female, M–male; time of the infection (**c**): colored by sampling week.

**Fig 2:**
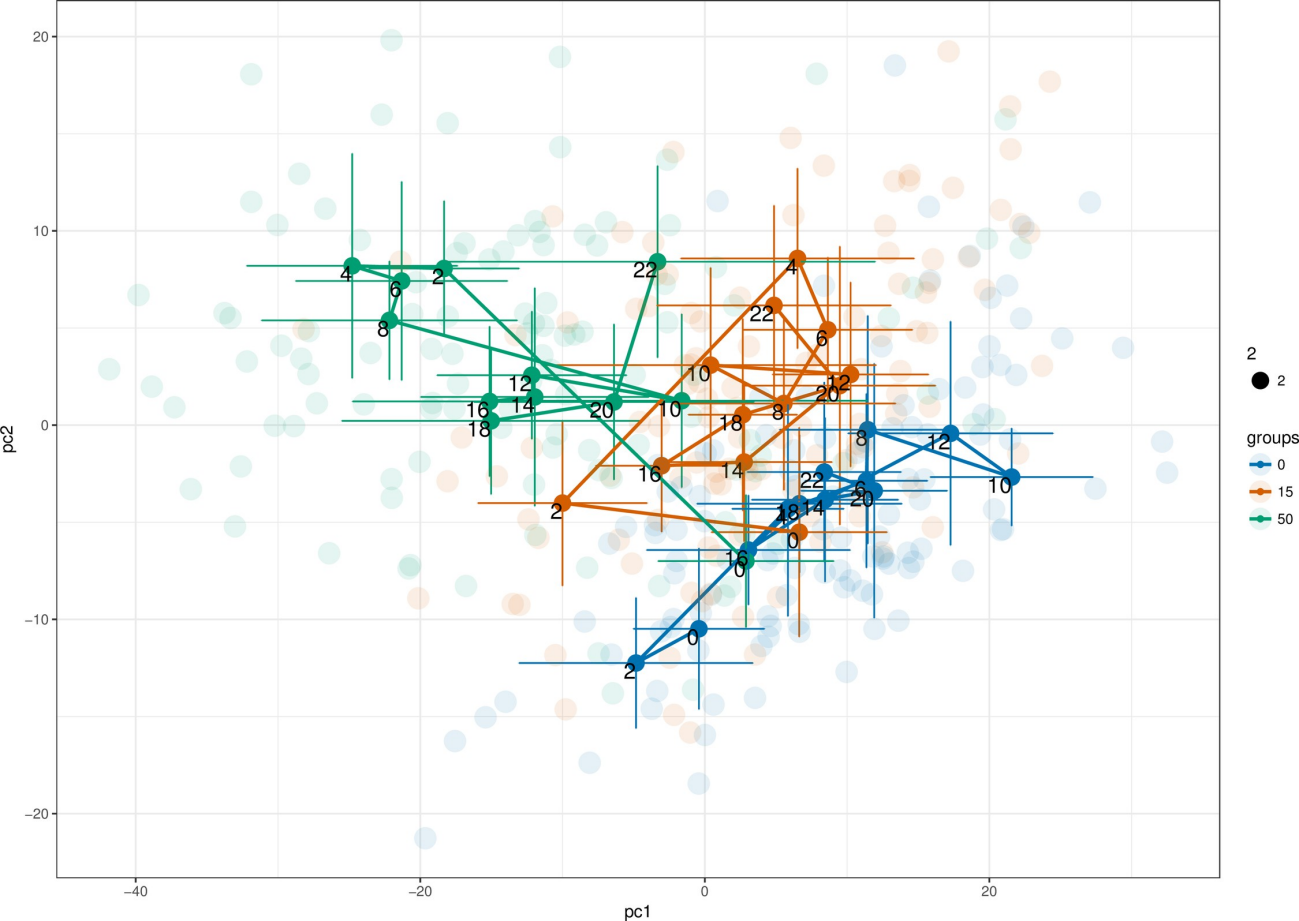
The geometric trajectory visualization of the PCA scores. Control group (blue); infected animals with 15 metacercaria per animal (orange); infected animals with 50 metacercaria per animal (green).

The worm burden and egg output were described in detail in the first part of the exploratory metabolomics of opisthorchiasis in the golden hamster [15]. To get an overview of the main sources of variance in the data we used principal component analysis (PCA). Fig 1 shows a PCA model built on the unit variance-scaled 1D NOESY data from all time points. The ten principal components (PCs) of the model cover 80% of the variance, with the first two components explaining more than 60% of the total variance. Contrary to our results reported for the urine samples, a clear trend related to intensity of the infection is visible already in the first two PCs (Fig 1A). No strong gender effect is observed (Fig 1B) and the influence of the sampling time appears surprisingly weak (Fig 1C). However, a geometric trajectory visualization approach [16] reveals the group-specific trends (Fig 2). The samples of both infection intensity groups and the controls collected at time point 0 (1 day before infection) are located close to each other. At the second and fourth weeks of infection, the difference between the experimental groups becomes maximal before gradually reducing towards the tenth week (Fig 2). From the tenth to the twenty second week the trajectories are somewhat random and remain within their own clusters.

### ANOVA-simultaneous component analysis

One of the main questions of our study is finding the metabolic features describing infection unfolding in time. There is only a limited number of statistical approaches for modeling a time-resolved metabolomics study design with several experimental groups [26]. One of them is ASCA (ANOVA-simultaneous component analysis)–a method based on the assumption that every variable is a function of all factors included in a study and uses analysis of variance for the analysis of the individual experiment factors and their interactions [23]. Thus, ASCA modelling estimates the contribution of every factor to the data variability. The ASCA analysis of our dataset shows that infection explains 33% of the variance in the data and as such is the most dominant factor, followed by time (18%); the interaction between the experimental factors (time and infection) explains 12% of the variance in the data. All factors and their interactions were significantly altered: the p-values for the infection and the time both were 0.001, while the p-value for them interaction was 0.0140.

**Fig 3:**
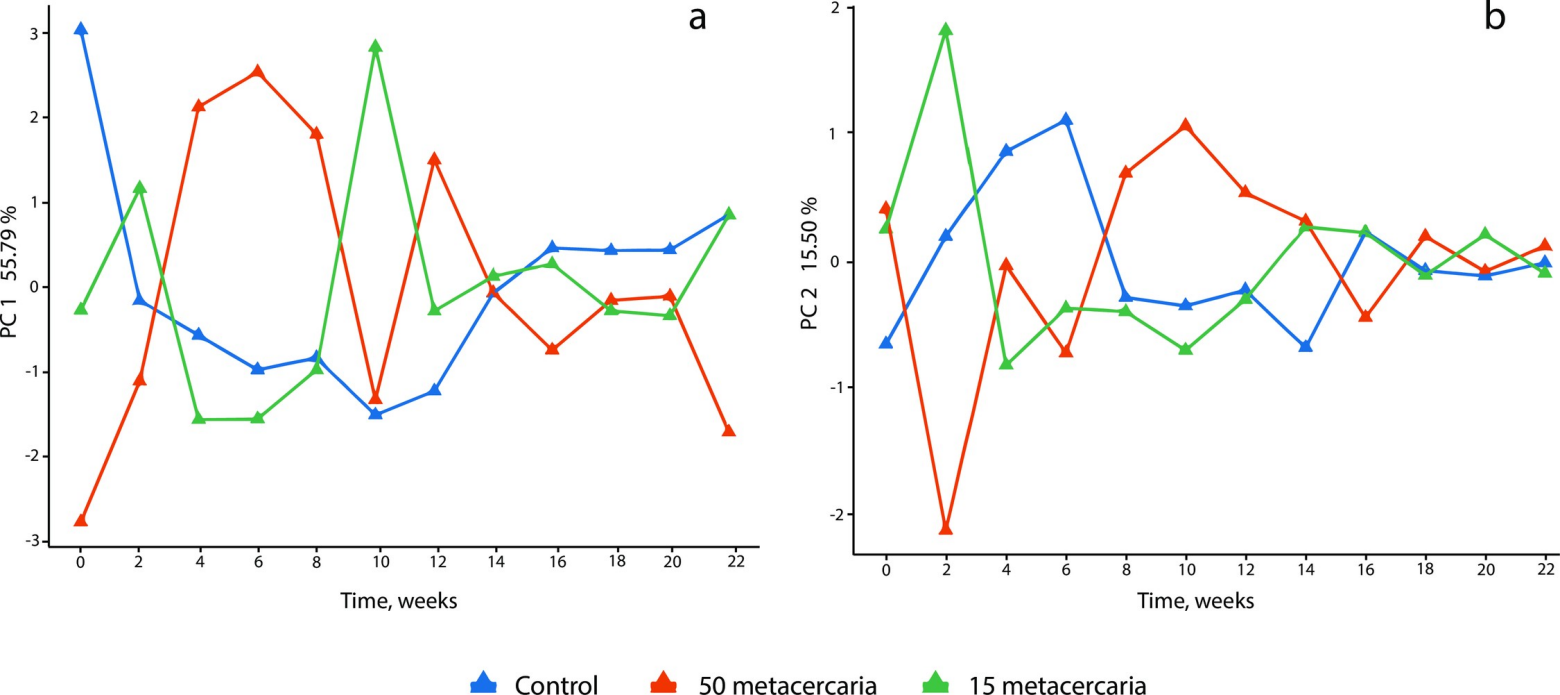
Scores plots of the on the first (a) and the second (b) components of ASCA model for the “Infection x time” interaction.

Fig 3 shows the score plots for a time*infection interaction submodel. The two components of the model explain 72% of the variance. The first component (56% of the variance) shows that the strongest metabolic changes are observed up to the tenth week (Fig 3A); in the second component (16%), the clear difference between the groups is seen only at the second week of infection (Fig 3B). Thus, the strongest metabolic response to the *O*. *felineus* infection is observed from 2 to 10 weeks post-infection, which is in agreement with the results of the study on urinary metabolomics of the *O*. *felineus* infected animals [15].

S1 Fig gives a visual summary of the relative contribution of the individual variables to the model explaining both infection and the time factors of the experiment. The variable importance is proportional to the value of its loading (Y axis); this way we can select a subset of influential variables and annotate them. Table 1 summarizes the annotated metabolites.

**Table 1:** Annotated variables selected on the basis of the ASCA model loadings.

δ, ppm	Annotation	Principal component
0.64–0.68	Cholesterol	PC2
0.82–0.88	Lipids CH_3_	PC1, PC2
0.96–1.00	Valine, Leucine, Isoleucine	PC2
1.08–1.12	Unknown	PC2
1.22–1.36	Lipids CH_2_, lactate	PC1, PC2
1.30–1.36	Lactate	PC2
1.94–2.02	Lipids (CH_2_)_n_-CH_2_-CH =	PC1, PC2
2.20–2.24	Lipids CH_2_-CH_2_-COOC	PC2
3.182–3.237	Lipids -N-(CH_3_)_3_	PC1, PC2
4.081–4.117	Lactate	PC2
4.24–4.28	Threonine	PC2
5.26–5.32	Lipids CH = CH	PC1

### Time trajectories of the significant metabolites

**Fig 4:**
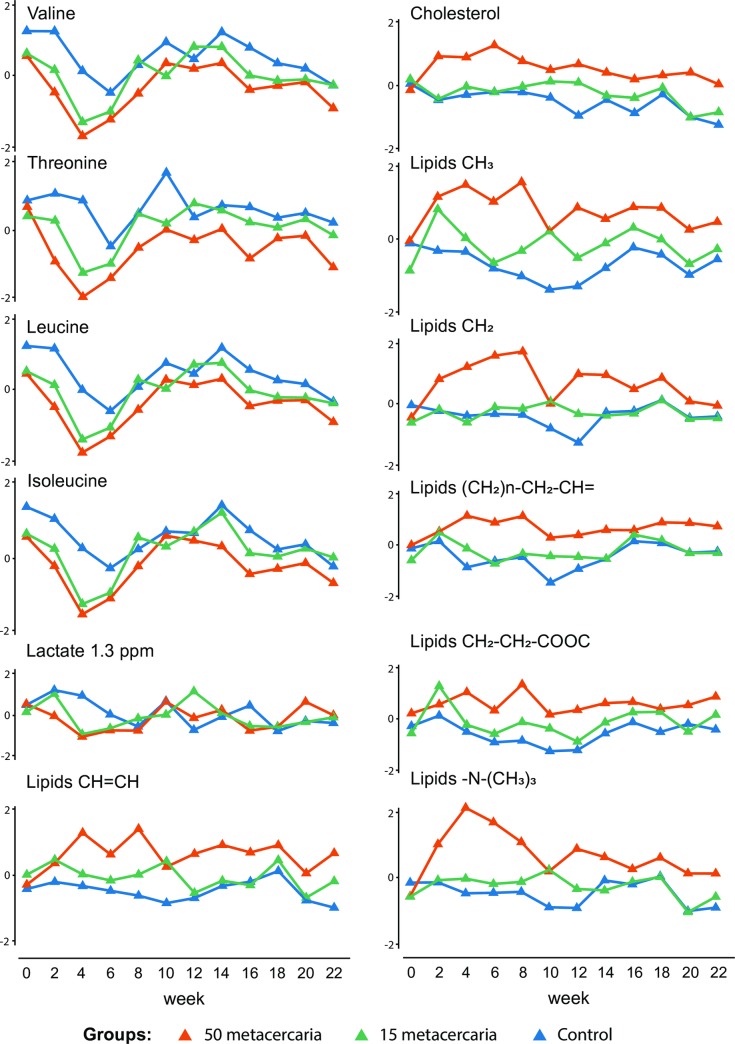
Spaghetti plots of the quantified metabolites selected on the basis of the two first PCs of the ASCA model, using the “infection*time” interaction. Blue–control group; orange– 50 metacercaria; green –15 metacercaria.

One of the most frequently used strategies of NMR data pre-processing is data reduction by binning, or more recently, adaptive binning. This approach was used for the current study as well. A bin is a narrow spectral region containing signals of zero, one, or only a few metabolites. On the other hand, a single metabolite can generate multiple signals or exceptionally broad signals that contribute intensity to multiple bins. Lipoproteins are an example of a plasma component with wide signals that cover several neighboring bins (see Table 1). Therefore, to obtain an additional validation of our findings and make practical use of the quantitative nature of the NMR we quantified the metabolites reported in Table 1. Amino acids and lactate were quantified using the 1D Carr–Purcell–Meiboom–Gill pulse sequence (CPMG) and the 2D J-resolved spectra (JRES) data [20]. For the lipoproteins and lipids, the corresponding regions of the diffusion-edited spectra were integrated. The 1D diffusion-edited experiment was developed especially for analysis of macromolecular assemblies without interference of the small molecules [25]. The integration of the relevant regions in the diffusion-edited experiment is probably the best possible alternative in absence of a true lipoprotein quantification model for hamster blood plasma. Fig 4 shows the time trajectories for the individual metabolites. In general, the trends are in agreement with the results of the ASCA time*infection submodel. To support the visual presentation with a statistical description we applied the multivariate analysis of variance (MANOVA) method to our quantitative data. The results summarized in Table 2 show that both the MANOVA model and its individual predictors (with an expedition of an unknown metabolite) indicated a statistically significant trend for time*infection interaction term.

**Table 2:** Results of the MANOVA analysis performed on the quantified metabolites.

MANOVA model metrics		Wilks λ	F	Pr(>F)
		0.474	6.640	2.2*10^−16^
Individual terms for interaction infection*time				
ID	Sum sq	Mean sq	F	Pr(>F)
Cholesterol	103.97	34.66	49.23	2.2*10^−16^
Lipids CH3	103.38	34.46	48.83	2.2*10^−16^
Valine	20.45	6.82	7.18	1.09*10^−4^
Leucine	20.34	6.78	7.14	1.15*10^−4^
Isoleucine	20.17	6.72	7.08	1.26*10^−4^
Unknown	7.71	2.57	2.61	0.05161
Lipids CH_2_	49.49	16.50	19.10	1.79*10^−11^
Lactate (1.3 ppm)	10.48	3.49	3.57	0.01435
Lipids (CH_2_)_n_-CH_2_-CH =	76.35	25.45	32.43	2.2*10^−16^
Lipids CH2-CH2-COOC	53.08	17.70	20.74	2.29*10^−12^
Lipids -N-(CH_3_)_3_	84.48	28.16	37.00	2.2*10^−16^
Lactate (4.1 ppm)	9.80	3.27	3.33	0.01971
Threonine	46.05	15.35	17.57	1.25*10^−10^
Lipids CH = CH	75.87	25.29	32.16	2.2*10^−16^

The resonances for quantification were selected on basis of the ASCA model.

## Discussion

The current manuscript is our second work dedicated to the description of the time resolved metabolic response to *Opisthorchis* infection in an animal model. The first part was focused on the metabolic changes detected in urine samples, the current one covers the infection dependent changes in the plasma samples. Both sets of samples (urine and plasma) were collected within the same study which was designed as a longitudinal study with two degrees of infection intensity (mild– 15 and severe– 50 metacercariae per animal) and a control group. The plasma samples were collected every second week until 22 weeks of post-infection; this time interval includes both infection response phases: the acute phase and the phase which roughly corresponds to a chronic stage. Our data show that the strongest metabolic response was observed between the 2nd and 10th weeks after the experiment: a time when the worms are reaching the bile duct where they are transforming into the adult form and starting the egg production [27]. It has been shown that the acute phase of *O*. *felineus* infection in humans is more pronounced than in *O*. *viverrini* and *C*. *clonorchis* infection and often is accompanied by diarrhea, abdominal pain, nausea and vomiting [28, 29]. Thus, it is important to mention that between the 2nd and 10th week of the experiment no diarrhea, loss of appetite, or loss of body mass was registered in the infected animals.

On a metabolic level the first ten weeks are characterized by rapid changes of amino acids and lipids which is in agreement with our report on urinary metabolomics [15]. The ASCA loadings plot (Fig 3) shows that the 4th week of the infection is the point where metabolic differences between the experimental groups are most pronounced. The egg production was recorded for the first time in the same week. One of the characteristic features of the metabolic response at this time point is a decrease of the blood plasma levels of the essential amino acids in the infected animals (Fig 4). It is worth mentioning that the observed decrease of the plasma levels of two essential amino acids isoleucine and leucine is associated with their increase in urine at 2nd and 4th weeks of infection [15]. The depletion of the essential amino acids (valine, isoleucine and leucine) during the acute stage of the infection was also described for *Echinostoma caproni* infection [30, 31]; the parasite-induced malabsorption is one of the possible physiological explanations of the effect [32]. A similar, but not identical trend (no data on isoleucine were reported) was observed in a co-infection model of *Necator americanus* and *Schistosoma mansoni* [33]. In the case of opisthorchiasis an interpretation based entirely on malabsorption is not applicable; the parasite is more likely to change the pool of the free amino acids in the circulation by affecting the liver function [34]. Threonine, another essential amino acid which plays an important role in protein and lipid metabolism, is also depleted in the infected animal by the fourth week (Fig 4). Interestingly, an increase of threonine was found in feces in a human study of *O*. *felineus* [35]. However, a confirmation of malabsorption and mechanism of influence on essential amino acids in case of *O*. *felineus* requires an additional study. Apart from the amino acids a short-time decrease of lactate in plasma samples of both infected groups (S1 Fig) was detected. However, the quantified data does not show clear trends beyond the 4th week post-infection (Fig 4).

Lipid metabolism also shows a clear response to the infection. However, it has to be mentioned that for hamster lipids NMR spectroscopy remains a sub-optimal technique. For instance, while cholesterol can be annotated and quantified from NMR data without a problem, the spectral regions of the physiologically important lipoprotein particles such as low density lipoproteins (LDL), very low density lipoproteins (VLDL), high density lipoproteins (HDL) are strongly overlapped. Recently an elegant solution for an accurate quantitative analysis of the serum lipo-particles by NMR was introduced [36, 37], sadly this platform is only available for human serum. For animal material we can only indirectly estimate their contribution to the lipid profile using the NMR peaks corresponding to the various chemical functional groups. Our data shows that for all lipid-related spectral areas which were selected on basis of the ASCA analysis (CH_2_, CH_3_ and CH = CH chemical groups and cholesterol) a very distinct time trend is observed for the severe infection group at the early weeks of the infection (Fig 4). From the 10th week of infection onward the trends for both infected groups become more alike but as a rule the group with the severe infection consistently shows the higher values.

Despite the fact that a shift in the lipid homeostasis during the acute phase of infection is well-documented for helminthiases, it is not easy to put our findings into the context of the existing reports. The main problem is the limited number of relevant reports, and the reports that exist use a variety of methodologies and experimental models. For instance, an NMR study on a mouse model of the *Echinostoma caproni* infection shows an increase of the lipid fraction related resonances in the infected animals. However, the maximum lipid increase was observed already at the 12th day of the experiment, which is much earlier that in our model (4 to 6 weeks) [30, 31]. At the same time, a mouse model of *Schistosoma japonicum* infection is characterized by a decrease of the lipoproteins from three weeks after infection [38]. There is no NMR-based study on *Opisthorchiidae*, but a report of Laothong et al. [39] where the cholesterol and lipoprotein particles in hamsters plasma were measured using the standard clinical chemistry methods is mostly in agreement with our data. Authors report an increase in cholesterol around one month after infections and a strong increase of LDL up to twelve weeks after infection. In the discussion they explain an increase in the LDL concentration as a reaction to leakage of vitamin E (alpha-tocopherol) from a damaged liver; a plausible interpretation which could be applied to our observations as well.

Even more difficult is to fit the observations obtained on the animal models to the data available on human material. The only clinical study on the lipids and lipoproteins in *O*. *viverrini* infection known to us [40] reports a significant decrease in cholesterol concentration for the group of the infected patients. It is very tempting to explain the differences in the observed trends as a result of the different technological approaches: colorimetric quantification of the cholesterol and NMR. Yet, we do not believe that this is the case. It has been shown that during the acute phase of an infection, the cholesterol level increases in the serum of the animals with low baseline values of LDL cholesterol (rodents) but remains stable in primates where the baseline values are higher [41–43]. Thus, the mechanisms controlling the lipid homeostasis in rodents and primates can explain the differences in the trends of cholesterol and lipoproteins between our data and the human studies.

### Conclusions

The time resolved study of the metabolic response to opisthorchiasis in blood plasma in the main lines agrees with our report on urine data. The response reaches its peak around the 4th week of infection and stabilizes after the 10th week. Yet, unlike the urinary data there is no strong effect of the gender in the data and the intensity of infection is presented in the first two principal components. The main trends of the metabolic response to infection in plasma are the transient depletion of the essential amino acids and an increase in lipoprotein and cholesterol concentrations.

## Supporting information

S1 Fig A visual summary of the relative contribution of the individual variables to the model explaining both infection and the time factors of the experiment. The variable importance is proportional to the value of its loading (Y axis); this way we can select a subset of the influential variables and annotate them.
(TIF)

S1 File NMR data. The file includes the original bin table of the NOESY data, JRES data on lipids, and quantified metabolites.
(XLSX)
