# Supplementary figures and images for: Plasma metabolomics of the time resolved response to Opisthorchis felineus infection in an animal model (golden hamster, Mesocricetus auratus)
Source: PLoS Negl Trop Dis. 2020 Jan 24;14(1):e0008015. doi: 10.1371/journal.pntd.0008015 (PMC7002010; doi:10.1371/journal.pntd.0008015)

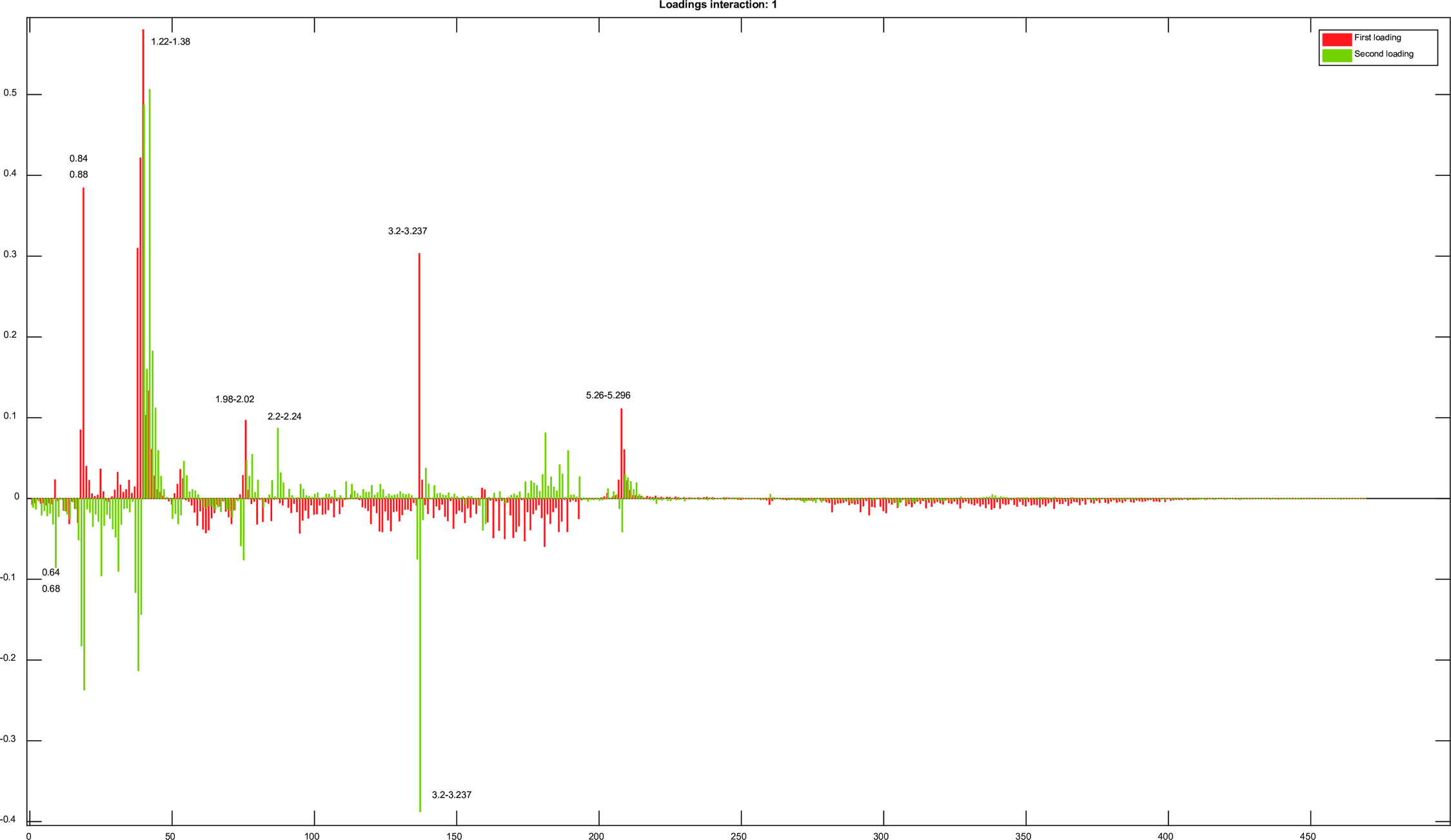

Supplement: S1 Fig — The variable importance is proportional to the value of its loading (Y axis); this way we can select a subset of the influential variables and annotate them. (TIF) [file pntd.0008015.s001.tif]
